# Investigating risk factors for urine culture contamination in outpatient clinics: A new avenue for diagnostic stewardship

**DOI:** 10.1017/ash.2021.260

**Published:** 2022-03-18

**Authors:** Patrick S. Whelan, Alicia Nelson, Christopher J. Kim, Christian Tabib, Glenn M. Preminger, Nicholas A. Turner, Michael Lipkin, Sonali D. Advani

**Affiliations:** 1 Division of Urology, Duke University School of Medicine, Durham, North Carolina; 2 Division of Infectious Disease, Duke University School of Medicine, Durham, North Carolina; 3 Duke Center for Antimicrobial Stewardship and Infection Prevention, Durham, North Carolina

## Abstract

Mixed flora in urine cultures usually occur due to preanalytic contamination. In our outpatient urology clinic, we detected a high prevalence of mixed flora (46.2%), which was associated with female sex and older age. Patient education did not influence the rate of mixed flora. Future efforts should target high-risk patients.

A positive urine culture in a symptomatic patient is considered the gold standard for diagnosis of a urinary tract infection (UTI). Urine culture plays a critical role in the management of urinary tract symptoms, hematuria, nephrolithiasis, and preoperative management of endoscopic urologic procedures.^1^ Preanalytic contamination of urine cultures during the collection phase can lead to falsely positive or mixed flora urine cultures, which in turn leads to inappropriate antibiotic use.^2–4^ Diagnostic stewardship interventions should focus on collection of urine specimens to reduce the occurrence of mixed urine culture results and provide opportunity for safe de-escalation of antimicrobials.^5^ We evaluated the incidence of and defined risk factors for mixed urine cultures in an outpatient urology clinic, and we reviewed the impact of patient education on the incidence of mixed urine cultures.

## Methods

### Design

This was a retrospective review of all urine cultures in an outpatient urology clinic at a large, academic medical center in Durham, North Carolina, from January 1, 2020, to October 31, 2020. This study was considered exempt from approval by Duke University Institutional Review Board (protocol no. 00103599). Only direct urine cultures were included; our laboratory does not perform reflex urine cultures.

### Definitions

Negative cultures were defined as urine cultures with no bacterial growth. Mixed urine cultures were defined by Duke University Microbiology Laboratory as the presence of 2 or more organisms when all organisms are nonsignificant (not a known uropathogen) or when 1 of the organisms is considered a significant uropathogen but is in lesser quantity (∼10-fold fewer) than the concentration of the nonsignificant organisms: for example, 1,000 colony-forming units per milliliter (CFU/mL) of significant compared with 10,000 CFU/mL of nonsignificant organisms.^3^

### Intervention

In July 2020, standardized patient education was initiated using an institution-approved instructions (Supplements 1 and 2) posted in patient restrooms and a urine collection handout (Supplement 3) for performing midstream voided urine collection. Prior to this, patients were given a urine collection cup and wipes without any standardized instructions on how to collect the urine sample. The baseline period was from January through July 2020, and the intervention period was from August through October 2020.

### Statistical analysis

Logistic regression analysis of the baseline cohort was performed to identify any risk factors: body mass index (BMI), age in decades, sex, *International Classification of Disease Tenth Revision* (ICD-10) stone disease diagnosis (codes N20.0, N20.1, N20.2 due to association with mixed flora) for mixed versus negative urine culture. A segmented regression (ie, interrupted time series) analysis was performed to estimate changes in monthly incidence of mixed urine cultures in the baseline and intervention periods.

## Results

During the study period, 1,306 urine cultures were reviewed. In the baseline cohort, 838 urine cultures were included, of which 372 (43.9%) were mixed urine cultures. The median age of patients was 62 years (interquartile range [IQR], 45.8–78.2); 40.6% of patients were female; and the median BMI was 28.7 kg/m^2^ (IQR, 24.8–32.9). Overall, 39% of female patients had a BMI >30 kg/m^2^. In the unadjusted analysis, the incidence of mixed urine cultures was higher in females compared to males (65.9% vs 29.7%; *P* < .0005), in patients with stone compared to nonstone diagnoses (54.8% vs 40.0%; *P* = .023), and in patients with BMI >30 kg/m^2^ compared to those with BMI ≤30 kg/m^2^ (47.9% vs 42.7%; *P* < .0005).

In multivariate logistic regression model, female patients had 16 times higher odds of mixed urine cultures, and increasing age was associated with 1.17 higher odds of mixed urine cultures per decade of life. BMI and stone diagnosis were not statistically significant (Table 1). The multivariable logistic regression model was statistically significant: χ^2^ (4) = 138.602 (*P* < .0005) and predicted 72.6% of mixed cases. The area under the curve was 0.787 (95% CI, 0.746–0.827).

**Table 1.** Risk Factors for Mixed Urine Cultures in Multivariable Logistic Regression Analysis

Variable	Odds Ratio	95% CI	*P* Value
Age, per decade	1.174	1.020–1.352	.026
Body mass index	1.012	0.980–1.046	.464
Sex, female	15.934	8.928–28.436	<.0005
ICD-10 stone diagnosis	1.311	0.817–2.160	.251

Note. CI, confidence interval; ICD-10, *International Classification of Disease, Tenth Revision.*

**Fig. 1.**
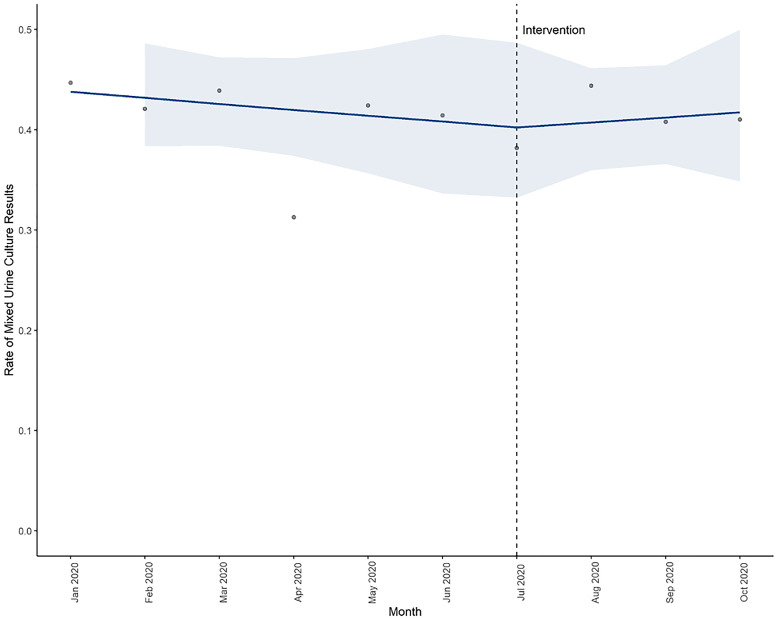
Interrupted time series model of monthly mixed urine cultures (with confidence intervals) in baseline and intervention periods.

In the intervention period, there was no significant change in the incidence of mixed urine cultures. The baseline trend was 0.99 (95% CI, 0.93–1.04; *P* = .62), and the level change with intervention was 0.97 (95% CI, 0.67–1.36; *P* = .88). The trend of mixed urine cultures remained stable; the slope change with the intervention was 1.03 (95% CI, 0.92–1.15; *P* = .65) (Fig. 1).

## Discussion

In our analysis, female sex and older age were associated with significantly higher odds of mixed urine cultures. Women have a shorter urethral length, which may predispose them to preanalytic contamination from vaginal flora, perineal skin, and perianal skin flora. Additionally, the female external genitalia often require greater retraction during collection than male external genitalia. Increasing age may lead to higher chance of preanalytic specimen contamination during collection or due to change in vaginal flora, especially for female patients. Our study is the first to identify increasing age as a risk factor for mixed urine cultures. Increasing BMI was also associated with higher odds of mixed urine cultures on univariate comparison, albeit not statistically significant in multivariable model. As BMI increases, so does the skin surface area necessary to retract and that may contact the urine during voiding. Similar results were obtained with contaminated urinalysis results in persons with obesity in a study conducted in the emergency department.^6^

Mixed urine cultures pose a diagnostic challenge to most clinicians. In some cases, clinicians may continue broad antimicrobial treatment or may order repeat urine cultures to confirm the diagnosis, thus increasing laboratory workload, causing processing delays. In other cases, clinicians may treat mixed flora as contaminants and may stop antibiotic treatment in high-risk patients risking infectious complications.^7^ The incidence of mixed cultures is increasing in inpatient and outpatient settings; thus, it is imperative to improve preanalytic practices.^4^

Several studies have sought to reduce mixed urine cultures with patient instructions, with limited success.^8,9^ Randomized collection techniques of clean and nonclean collection in asymptomatic women have not shown difference in urine culture contamination.^10^ In these studies, contamination was <40%, and our results were ∼46% mixed urine cultures. Our study highlights that the major risk factors for mixed urine cultures include sex and age, which are not modifiable. Hence, an instructional patient handout focusing on reducing preanalytic specimen contamination by improving collection techniques is unlikely to show a significant impact. In this situation, in addition to optimizing transport and storage, it is important do the following: (1) identify high-risk patients, which includes older females with higher BMIs; (2) order urine cultures only when clinically indicated; and (3) offer urine specimen collection by straight catheterization.

Our study had several limitations. This retrospective study was performed at a urology clinic in a single, large, academic medical center. We did not differentiate between preoperative cultures and urine cultures in symptomatic patients. However, it is likely that our ICD-10 codes captured the primary diagnosis rather than the diagnosis for which the urine culture was ordered. Although we did not have access to data on transport or processing time of urine cultures, these processes are independent of patient demographics. If a laboratory- or transport-related issue was present, we would not expect to see patient-specific traits like sex act as such a strong predictor.

In conclusion, we detected a high baseline prevalence of mixed urine cultures in the outpatient urology clinic. Female sex and older age were significant risk factors. Patient education did not appear to change the overall incidence of mixed cultures, due to the nonmodifiable nature of our risk factors. Future efforts should focus on targeted strategies directed toward our high-risk patients (ie, older women) to reduce preanalytic contamination of urine cultures, which is an important stage of diagnostic stewardship.^5^

## Supplementary material

For supplementary material accompanying this paper visit https://doi.org/10.1017/ash.2021.260. click here to view supplementary material
