# Supplementary material for: Investigating risk factors for urine culture contamination in outpatient clinics: A new avenue for diagnostic stewardship
Source: Antimicrob Steward Healthc Epidemiol. 2022 Mar 18;2(1):e29. doi: 10.1017/ash.2021.260 (PMC9016366; doi:10.1017/ash.2021.260)

Directions for obtaining a clean catch urine specimen

Patient Education

Directions for men

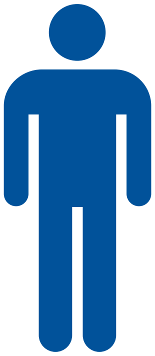

1. Wash hands

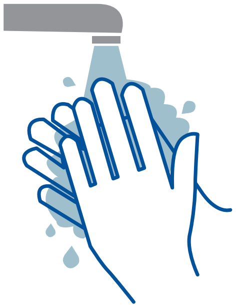

2. Open cup

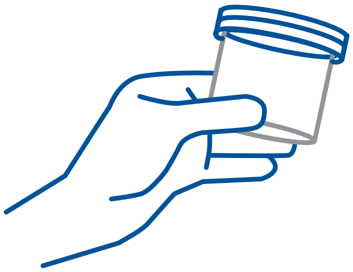

3. Open wipes

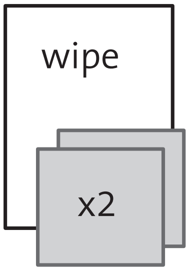

4. Clean once with each wipe

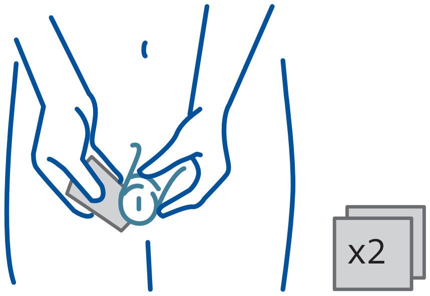

5. Start to urinate in toilet

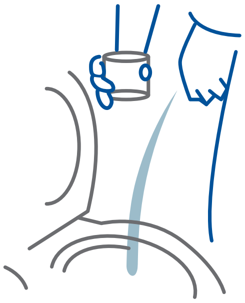

6. Place cup in urine flow

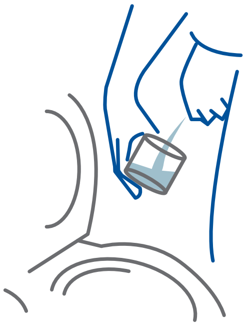

7. Give to nurse

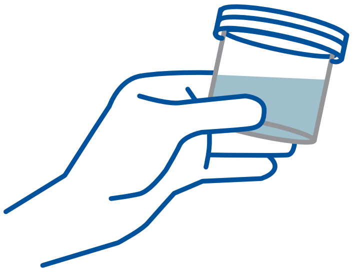

Supplement: Supplementary file 1 [file ashsup.zip › S2732494X21002606sup001.pdf]
